# Virtual Visits in Home Health Care for Older Adults

**DOI:** 10.1155/2014/689873

**Published:** 2014-11-20

**Authors:** Anne Marie Lunde Husebø, Marianne Storm

**Affiliations:** Department of Health Studies, Kjell Arholms Hus, Universitetet i Stavanger, 4036 Stavanger, Norway

## Abstract

*Background*. This review identifies the content of virtual visits in community nursing services to older adults and explores the manner in which service users and the nurses use virtual visits. *Design*. An integrative literature review. *Method*. Data collection comprised a literature search in three databases: Cinahl, Medline, and PubMed. In addition, a manual search of reference lists and expert consultation were performed. A total of 12 articles met the inclusion criteria. The articles were reviewed in terms of study characteristics, service content and utilization, and patient and health care provider experience. *Results*. Our review shows that in most studies the service is delivered on a daily basis and in combination with in-person visits. The findings suggest that older home-dwelling patients can benefit from virtual visits in terms of enhanced social inclusion and medication compliance. Service users and their nurses found virtual visits satisfactory and suitable for care delivery in home care to the elderly. Evidence for cost-saving benefits of virtual visits was not found. *Conclusions*. The findings can inform the planning of virtual visits in home health care as a complementary service to in-person visits, in order to meet the increasingly complex needs of older adults living at home.

## 1. Introduction and Background

According to the United Nations [1], developed countries are facing a global, demographic challenge from their growing population of older people. Among people 80 years or over, the average annual growth rate is twice as high as it is among people 60 years or older [1]. Although many elderly people stay healthy, the increase in life expectancy results in a higher prevalence of chronic diseases, disabilities, and/or reduced activities of daily living (ADL) skills. These issues reduce the ability to take care of one's own needs and increase the need for assistance from family members or informal caregivers, as well as health care services for older adults. An anticipated lack of health care professionals adds to the challenge. Recruiting and retaining highly skilled staff in care for the elderly is becoming difficult and highlights the issue of providing adequate care [2]. There is also a growing interest amongst older individuals to age at home rather than in a health care facility, and living at home is associated with higher quality of life, dignity, and independence [3]. The aging population aging at home and qualified staffing together constitute a public health concern; in addition, they are of special concern to home health care services in terms of organizing and providing safe and high-quality services. A recent review reports on differences between European countries in policies on home care and on the organization and availability of home care services [4]. Many European countries will need to reconsider their service delivery systems, based on demographic developments and financial constraints. The reconsideration would probably entail attention to organizational change, staff by-in and innovative approaches to health care service delivery.

European countries are not alone in facing these challenges. In the USA, home care for the elderly is based on efforts of family caregivers [5], and concerns have been raised as to whether informal health care can accommodate the complexity of an aging nation [6]. Finding new solutions to meet the needs of home-dwelling older people has advanced the development and implementation of assistive technology as part of service delivery in the home.

The term “home telehealth” includes application of telehealth innovations such as interactive audio/video transmissions, videophone technology, monitoring of the patient's condition, and physiological parameters such as oxygen saturation, pulse, and respiratory rate in the home environment [7, 8]. Home telehealth delivered as* virtual visits *includes the use of real-time audio-visual communication devices, in this review acknowledged as videoconference by the use of videophones, personal computers/laptops, or the TV screen [9]. This means that a traditional in-person visit from the care provider is replaced with a virtual visit, used to assess a patient's health status, monitor medication routines, demonstrate procedures, and provide social contact [10]. A virtual visit allows for a natural and interactive communication form that can build a trustful relationship between the patient and the nurse [11]. Virtual visits have been associated with patients feeling secure and satisfied with health care information services [10]. The challenge often lies in the user-friendliness of the technical communication devices [11]; technical problems with communication equipment [12]; and meeting patients' preferences and care goals [10]. Virtual visits have also been problematic when the service user has a cognitive impairment [13].

Previous literature on home telehealth has evaluated the economic value of telehealth, considered the integration of health-enabling technology in standard health care services, and assessed the field of home telehealth services for older people suffering from chronic diseases [2, 10, 12, 14]. Moreover, it includes a variety of technical devices, such as audio-video communication systems for monitoring of health-related conditions and smart-house technology, including devices used in health telecare. There is a lack of focus on possible tensions in the relationship among the technology, the patient, and the nurse. Sävenstedt et al. [15] have noted a dichotomy between dignified (i.e., person-centered) and undignified care, claiming that implementation of technology in elderly care can promote both dignified and undignified care. The variety in telehealth approaches and methods implemented in home care settings makes it difficult to assess the pros and cons of two-way, real-time, audio-visual communication in the delivery of daily and/or weekly home care health services to the elderly. Thus, our study reviews the published research on care content and utilization of virtual visits and in particular how old patients and the health care providers use a virtual visit and how they experience it.

## 2. Methods

An integrative review of both quantitative and qualitative research was performed as outlined by Whittemore and Knafl [16]. The inclusion of studies using multiple methodological approaches can broaden the understanding of the impact of virtual visits in future home health care and aid in planning home telehealth practices.

### 2.1. Search Strategy

A literature search was carried out prior to the integrative review. The literature search was performed in the electronic databases Cinahl, Medline, and PubMed for papers published between January 2003 and April 2013. The following search terms were used:* video conferencing *OR* telecommunications *OR* telehealth *OR* ehealth *OR* telecare. *During the search in all three databases the search terms were combined with [AND]* elderly *OR* old *OR* aged *OR* senior *OR* frail elderly *[AND]* home care *OR* home care service *OR* home-based care *OR* community health service *OR* community dwelling*. The terms* videoconferencing*,* telehealth*,* ehealth*,* aged*, and* elderly *are MeSH words in the PubMed database, while* home care*,* home care services, videoconferencing, telehealth, *and* telecommunications *are Cinahl headings. In addition to the literature searches in the electronic databases, reference lists of both the included studies and of previously published reviews on the subject were examined for eligible studies. Reference tracking was conducted by consulting a telehealth expert.

### 2.2. Study Selection

**Figure 1:**
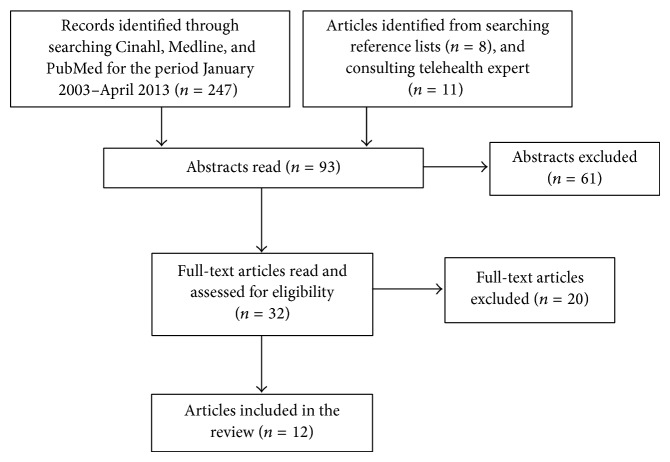
Flow diagram of study selection process.

Titles, abstracts, and full-text articles were read independently by the two authors (AMLH and MS). Consensus was reached in cases of uncertainty or disagreement on a study's eligibility. Studies included in the review were written in English, had been subject to peer review, and were published in scientific journals and focused on virtual visits as part of home care health services for the elderly. Thirty-two full-text articles were assessed for eligibility. Of these, 12 were included in the review. The search strategy and selection process are presented in a flow diagram in Figure 1.

Excluded studies did not meet the inclusion criteria and focused on either the use of audio-visual communication between elderly living in a nursing home and family members or the use of videoconferencing for communication between service providers only, videoconferencing as a peer-driven intervention, videoconferencing as part of cancer, palliative or mental health care, or videoconferencing between the patient and his or her physician's office.

### 2.3. Data Abstraction and Analysis

The analysis process is presented in Table 1. Analysis of the data was carried out to obtain a comprehensive and unbiased interpretation of the primary sources [16]. All data addressing the research theme of virtual visits in a home health care setting were independently extracted from the included articles by the two authors (AMLH and MS). After a constant comparison of the data material, looking for patterns and main themes, consensus was reached. The data were classified in terms of* study characteristics*,* service content* and* utilization*,* patient experience,* and* health care provider experience *(Table 1, step 1: data reduction). Next, the analysis and synthesis processes continued by reducing the classifications into subgroups (Table 1, step 2: data comparison). This was done to be able to organize and compare the primary sources on specific subjects and relationships [16].

### 2.4. Quality Assessment

Evaluation of quality in integrative reviews is difficult due to the inclusion of studies with multiple methodological approaches [16]. However, the authors conducted a thorough review of the quality of the quantitative studies, focusing on study design (e.g., sample size, randomization process). The question of “quality” in qualitative research is debatable, and rigid checklists can be inappropriate to use. This might complicate the attempt to synthesize and summarize study quality and constitutes a source of heterogeneity [17].

## 3. Results

### 3.1. Study Characteristics

Characteristics of the 12 included studies are presented in Table 2. The studies were published between 2003 and 2012, with a peak in 2007-2008. Six of the studies were conducted in North America, five in European countries, and one in Australia. Four of the studies were randomized controlled trials, with study samples ranging from 20 to 218 subjects [20–22, 29]. One of the studies combined a descriptive design with quantitative measurements [25]; three applied a descriptive design with semistructured interviews [19, 26, 28]; one combined qualitative interviews with an observational study [23]; one was a longitudinal study [24]; one was a cross-sectional study [18]; and one used a quasi-experimental design combined with qualitative data [27]. Elderly individuals (>65 years) were represented in all of the studies, while seven studies also included health care providers [18, 19, 21, 23, 26, 28]. One study included informal caregivers as study participants [27]. All of the studies were conducted in a home care setting.

### 3.2. Content and Utilization of Virtual Visits in Home Health Care to the Elderly

Content and utilization of the virtual visits differed substantially among the reviewed studies. Four dominant themes on content of the virtual visits were identified: psychosocial and educational interventions to reduce loneliness and increase activity levels, observation and support to enhance medication compliance, support and monitoring in chronic disease management, and follow-up service and monitoring medication self-administration to reduce readmissions to hospitals or long-term facilities. In most studies [19, 20, 24, 26–28] the virtual visit was delivered on a daily basis. In four studies [21–23, 29] the virtual visit service was delivered weekly, and two studies [18, 25] do not mention service delivery frequency. Seven of the studies report virtual visits being delivered in combination with in-person visits [19–22, 28, 29].

Prevention of social isolation and increase in social activities were studied outcomes in three studies [18, 24, 26]. Arnaert and Delesie [18] evaluated a videophone service for homebound elderly people, focusing on the relationship between the care and social support received and on health outcomes. The virtual visit consisted of psychosocial support and educational interventions and was delivered by nurses. Principles of social contact, safety and care mediation guided the visits. In Savolainen et al. [26], the ACTION study was designed to encourage social networking and to serve as an information center for elderly service users and their informal caregivers. A call center with experienced nursing staff operated the videophones, helping the families to manage their situation while offering support and practical information. Van Der Heide et al. [24] report on the “Good Morning, Good Evening” service, delivered by nurses and aimed at the patients' need for increased social contact and activity. The service was delivered by trained care workers.

Medication safety was the focus in four of the studies [21, 24, 27, 28]. Demiris et al. [21] found that the nurses spent 13% of their communication time in a virtual visit assessing a patient's medication compliance. In Smith et al. [27] the home telehealth service was designed to deliver medication management by virtual visits to elderly home-dwelling patients with mild cognitive impairments. The patients' compliance with medication was monitored, and support was offered by means of videophones. Support of medication compliance was also included as part of a telehealth service in the study of Van Der Heide et al. [24]. Wade et al. [28] studied the efficiency of using videophone to monitor medication compliance in elderly patients, in which two of the cases were part of directly observed tuberculosis treatment. As in Smith et al. [27], the researchers acknowledge limitations in the use of this service due to the physical and cognitive impairments of elderly patients.

Support and monitoring of a chronic medical condition in elderly people receiving home care was the primary objective of the home telehealth service in four studies [19, 21, 23, 25]. Observing and assessing the patients' clinical status was performed 42% of the time during the virtual visit and was by far the topic/area on which the nurses spent most of their visual communication [21]. Virtual visits delivered by videophone were used for observations of the patients' condition, for real-time transmission of physiological data [19, 25], and for advising the patient in disease self-management [19].

### 3.3. Elderly Service Users' Experience of Virtual Visits

Six of the reviewed studies provided information on patients' experience by means of interviews and/or questionnaires. Decreased loneliness and enhanced psychosocial contact were important results in half of the studies [18, 20, 21, 24, 26, 27]. The patients reported that the visual contact with the health care workers made them feel cared for and created a sense of connection. The patients reported feeling less isolated and indicated an increase in their social activities. The virtual visits also made the patients feel safe and secure [22, 24, 26]. Bowles et al. [20] reported that the patients who received a virtual visit reported significantly higher levels of personal contact than did patients receiving the usual home care visits. In the wake of decreased isolation and increased social contact, the patients experienced less melancholia and improved memory and attention, especially if they had physical and mental health problems [18].

Virtual visits can be used to monitor patients' medication compliance and to support them in medication self-administration. Although the two studies [27, 28] investigating a virtual visit service in medication surveillance were limited to elders with cognitive impairments, the results may be generalized to other patient groups. Valuable results from the studies are maintained competence and security in self-administration of medication [24, 27] and opportunity for medication review from service providers, thereby reducing the risk for adverse events related to self-administration of medications [28].

Evaluation of the quality of home telehealth showed that patients receiving a virtual visit and audio-visual communication with health care providers were more satisfied with the access and flexibility of the service than were recipients of standard home care [20, 22, 29]. They were confident that the nurses could monitor and assess their health status through the video communication system and provide them with good quality care. Finkelstein et al. [29] noted that being able to schedule videoconferences on topics that they had chosen was of great value to the elders living at home. Obtaining a rapid response from the nurses and taking part in planning the care were mentioned as positive features of the technology used to manage the treatment of chronic leg wounds [23]. The majority of the studies reported on user-friendly technology and noted minor challenges in the implementation and use of the communication devices. One exception is the study by Arnaert and Wainrigth [19] in which elderly patients with chronic obstructive pulmonary disease complained that it was difficult to manipulate the medical devices and to use the computer mouse and buttons.

Home telehealth by virtual visits as part of follow-up from health care services and after hospitalization shows mixed results for hospital admissions and readmissions. In the study by Smith et al. [27], informal caregivers expressed that monitoring the patient's medication compliance also prevented the elderly person from relocating to a nursing home. Finkelstein et al. [29] reported that patients who received telehealth had fewer hospital admissions than did patients with usual home health care. Bowles et al. [20] found that the numbers of readmissions at day 30 and at more than six months were lower for patients in the telehealth group but results were not significant.

### 3.4. Home Health Care Nurses' Experience in Delivering Virtual Visits

The experience of the nurses who delivered health services through virtual visits was studied in five of the studies [19, 21, 23, 25, 26]. In general, the nurses evaluated the virtual visit service positively. It simplified their efforts in teaching and informing the patients and thus became a source for knowledge transfer, which increased the patients' possibilities of self-care. The technology made it possible to deliver continuous and coordinated care, preventing relapses into poor health. The nurses reported that the telehealth was more flexible and easy to arrange, and the communication became trusting and more personal through the videoconference system. Another interesting result is that the nurses in the study of Savolainen et al. [26] found the virtual visits to aid in the prevention of colds and infections.

The nurses had some concerns in the use of virtual visits in home care for elders. One concern was for possible invasions of patients' privacy. In addition, Mair et al. [25] noted the nurses in their study were less comfortable with the videophone than their patients were. The nurses were concerned about not being able to observe the patients' health condition through the video screen. None of the studies revealed concern amongst nurses regarding the technology replacing personal contact or making the service impersonal and task-oriented.

## 4. Discussion

This integrative review focuses on home telehealth in community health care services for older adults. It provides a systematic overview of the content and utilization of using virtual visits in the delivery of health care services to the home-bound elderly. It also explored the elderly service users' and nurses' experiences with using virtual visits as part of home health care services. The reviewed studies showed a variety in content and purpose of virtual visits in home care services to older adults and were within the scope of the review. Thus, one cannot rule out the possibility of virtual visits being appropriate for services other than those identified in this review.

One important finding from this literature review is that virtual visits can reduce social isolation among the elderly. Research shows that one-third of people over 65 years of age live alone, and living alone is associated with functional impairment, poorer health, and social isolation [30]. Social isolation is a multidimensional concept that can be understood as the lack of social support in both quantity and quality [31]. Virtual visits involve real-time communication and contact between service users and health care providers by means of videophone or other technical devices. It is a personal meeting although the contact is not in person. If well-planned and used in a purposeful way, any audio-video communication service could counteract social isolation and increase social inclusion. Ageing at home is the preferred mode of care by the elderly population in many countries and is associated with independent living [4]. Research has identified fear of losing one's independence as a common concern amongst older people [32]. Use of technology at home is suggested to lower older people's sense of dependence on other people in their daily life and care [33]. The challenge for administrators of health care services is in creating efficient and high-quality health care services while ensuring dignified care that will promote patient autonomy and integrity [15].

Wade et al. [12] note that real-time video communication has been introduced to reduce costs and to increase access to health care services. Our results imply that real-time video communication in a home health care setting can contribute to reductions in hospital admissions and postponement of transfer to nursing homes, but the results are not conclusive for cost savings [20, 27, 29]. Bowles et al. [20] reported that telehomecare patients were readmitted less often than usual care patients for all causes; the results were not statistically significant. The economic analysis of 36 articles using telehealth technology and real-time video communication by Wade et al. [12] is also inconclusive. Rural service delivery using real-time video communication was reportedly more expensive for health care services, but cost saving for patients who do not need to travel. It is also suggested that video communication could be cost-effective in home care, especially when utilizing low-cost equipment such as the patient's television or small video units [12].

In this review, one study reported virtual visits to be time saving for nurses when administering medication; nurses saved as much as 60% of the cost compared to a standard field visit [28]. By fostering medication compliance by identifying a patient's barriers to compliance, and including reinforcement and reminders, virtual visits can contribute to better self- management and increased patient satisfaction [21]. Virtual visits might be particularly useful and cost-effective for patients who are not in need of any support or nursing care aside from medication compliance. Self-administration of medication is also related to the issue of patient safety. Elderly people can encounter difficulties in adherence to medication management and low compliance can put the patient at risk of health deterioration [34]. Virtual visits can make it possible for nurses to monitor patients' medication compliance and also to enhance the patients' knowledge and understanding of their medications and to reduce the risk of confusion about their purpose and administration.

One unexpected finding was that few studies report a concern of reduced quality of care when the in-person encounter in the older person's house was replaced with a virtual visit and remote communication with health care professionals [15]. Botsis and Hartvigsen's review [10] showed that even though the patients accepted the technology and felt secure and confident about the nursing assessment, they were afraid of losing the face-to-face contact with their health care providers. In Pols' study [35], nurses were especially worried that implementation of virtual visits in home care settings would result in impersonal care, making the nurses unable to take good care of their patients. In the present review, both patients and nurses were generally pleased with the quality of the communication through videophones; in only one study, nurses expressed concern that the technology could negatively affect the quality of nurse-patient communication and jeopardize the nurses' ability to observe changes in their patients' health [25]. The majority of the studies combined virtual visits with in-person visits, which might explain the high levels of patient and nurse satisfaction in this review. Implementation of telehealth has proven successful when nurses ensure the link to clinical practice by keeping up their traditional nursing style [35]. This is in line with the conclusions of Demiris et al. [21], claiming that not all nursing activities are suited to virtual visits and that choosing this service in a home care setting should be based upon the patient's medical and clinical condition and upon the extent of his or her care needs.

Only one of the reviewed studies [26] included informal caregivers as informants in their research. Here, interviews with noncohabiting caregivers provided qualitative data on opinions of telehealth services. Most elderly people expect to be supported and cared for in their homes by their closest relatives, and not by professional caregivers [32]. Care provided by family members, referred to as “invisible care,” is equivalent to one year of work hours provided by a health professional [36]. Some countries expect a substantial increase in the hours of average family eldercare (that is caring for an elderly family member) from 29 to 41 hours per week by 2030 [37]. Thus, it is crucial to include informal caregivers in future research on the use of videophones as part of home telehealth.

The reviewed studies revealed limited attention to training of the older users and nurses prior to, and during use of, the videophone technology in the person's home. To facilitate use of home telehealth and virtual visits, health care professionals need to focus on the benefits of home telehealth, in particular the possibility for closer follow-up from health care services and monitoring of the health condition [20, 33]. To succeed in changing health care professionals' embedded work practices Grol and Grimshaw [38] have proposed a five-step model: (1) assess their readiness to change, (2) assess barriers to guidelines use, (3) determine the appropriate level of intervention, (4) design the dissemination and implementation strategies, and (5) evaluate the implementation strategies. The model builds on several theoretical approaches, including an educational one. Transferred to implementation of home telehealth, the educational approach suggests that leaders of health care services use learning processes to motivate their workers to change by involving them in the planning, implementation, and evaluation of the technology.

Successful implementation and use of technology depend on a design that is usable and useful for elderly home-dwelling people in what Rogers and Fisk [39] term the* human factors principle*. Experiences with and acceptance of technology and home telehealth amongst the elderly may vary [20, 40]. Barriers reported are the elderly's lack of interest in technology use, worries about the cost, and being too infirm to use home telehealth and new technology [20, 33]. To meet the needs and preferences of these older service users, videophones need a design that is user-friendly, flexible, reliable, and aesthetic [13]. There is also a need for research into the design of appropriate training programs and learning skills needed to succeed with virtual visits in a homecare setting.

### 4.1. Limitation

The present review is subject to limitations. Although our literature was comprehensive, the challenge in the field of home telehealth is the variety of terms used and the interdisciplinary character of the field (research performed in fields such as medical informatics, engineering, and medical and caring science) [2]. In addition, searching other relevant databases (e.g., Scopus) might have provided a larger number of eligible articles. There is a risk that relevant papers have been overlooked. This review confirmed earlier research, pointing towards the lack of large-scale, controlled trials for hypothesis testing and also longitudinal studies evaluating the impact of technology from a long-term perspective [41]. Several of the studies included in this review can be considered as small-scale implementations of virtual visits and testing of audio-visual communication between elderly service users and health care provider. In addition, five of seven studies [20, 21, 23–25] that collected quantitative data by questionnaires used scales developed for the purpose of the particular study. All but one study [20] report inadequately on scale development and testing, making it difficult to review those scales' reliability and validity.

Performing an integrative review allowed for the inclusion of studies with substantial methodological diversity. Although considered a strength of integrative reviews by contributing to a broader perspective on the studied phenomenon, we acknowledge that sampling and comparison of heterogeneous studies might lead to the possibility of biased conclusions [17]. The results should thus be interpreted with caution.

## 5. Conclusion

The review identified virtual visits as having the clear advantages of enhanced social inclusion and less loneliness for the elderly patient and also providing support and guidance in the patient's self-management of medication. Both service categories could help postpone admission to long-term facilities or the need for substantial in-home care. However, the studies in this review do not provide strong evidence for cost savings of virtual visits as part of elderly home health care services. Thus, the review does not support the claim that virtual visits can be more than a supplement to traditional in-person home care visits.

The fact that both patients and nurses, in general, found the technology satisfactory might be attributable to the most frequent service models applied in the studied reviews, combining virtual visits with ordinary in-person visits. The literature shows a duality in using technology in the home care services, warning against eliminating in-home visits because of the advantage of virtual visits [10, 14]. This highlights the discussion of whether virtual visits can compensate fully for in-person visits, especially concerning the social aspect of the service. The challenges lies in finding the optimal fit between home telehealth and a patient's specific health concerns [41].

We believe that this review could inform both administrators and clinicians working in community health care services on how virtual visits can be integrated into home care services for older adults. Moreover, the review can inform further development and implementation of virtual visits in a home health care context.

## Figures and Tables

**Table 1:** Data abstraction and analysis^*^.

Step 1: data reduction^*^	Step 2: data comparison^*^ (a sample)
Study characteristics	Research design
	Health care setting

Service content and utilization	To reduce loneliness
	To enhance medication compliance

Patient experience	Decreased loneliness and enhanced psychosocial contact
	Satisfaction with the access and flexibility of the service

Experience of home health care nurses	Simplified teaching and informing the patient
	Concerns for patient privacy

^*^Adopted from Whittemore and Knafl (2005) [16].

**Table 2:** Overview of included studies (*N* = 12).

Authors (year)	Aim	Design	Participants	Virtual visits program	Outcome measures	Findings
Arnaert and Delesie (2007) [18]	To develop measures of change in homebound elderly peoples' functioning and identify characteristics of individuals whose functioning improves with videophone (VP) nursing care	Evaluation of a home telehealth program using eight different assessment scales	71 elderly with mean age of 72, 70% living alone and 76% receiving formal care	Psychosocial support and educational interventions	Loneliness Scale Geriatric Depression Scale SF-15 Social Network Scale Activities of daily living Instrumental activities of daily living Medical Outcome Study SF-36 Geriatric Center Moral Scale	The VP nursing care reduced social loneliness and melancholia and improved social activity and memory. No improvement in ADL was reported. Frequency of VT calls showed a significant and positive association with change in general health functioning in individuals with small social networks and reduced family support. Time watching television, poor social network, and age >75 years were significantly associated with improved self-perception

Arnaert and Wainrigth (2008) [19]	To uncover challenges involved in implementing remote monitoring and interactive video technology for elderly patients with chronic obstructive pulmonary disease (COPD)	Feasibility study applying a descriptive and evaluative design	Three nurses 34–58 years of age. Three patients 56–83 years of age with COPD as their primary diagnosis	Daily virtual visits from a telehealth nurse to explore patients' need of nursing interventions following hospital discharge	n.a.^*^	Reported challenges in running the home telehealth (HTH) were related to team performance, nurse training, patient recruitment, and technical issues with installation and use of HTH. The nurses received an average of 11.5 hours of HTH training prior to the implementation of HTH. The patients experienced difficulties in troubleshooting technological issues and handling medical devices

Bowles et al. (2011) [20]	To compare a home telehealth (HTH) intervention for elderly patients following hospital discharge for heart failure to usual skilled home care	Randomized controlled trial	Two hundred and eighteen heart failure patients: 102 in intervention group and 116 in control group. Six-month follow-up of study participants	The intervention group received a combination of in-person and HTH daily monitoring and intermitted video visits. Control group received usual care. The protocol defined a minimum of four video visits, daily use of home device, and 5 in-person home visits	Patient satisfaction (questionnaire^a^) Health care utilization (home care agency records, and health system database), access to care (home care agency records)	Of the 101 patients in the HTH intervention, 36 did not receive or wanted to receive any HTH. Younger patients were more likely to accept the technology. Access to care, more in-person contact, and satisfaction were significantly higher for HTH patients. No significant differences between intervention and control group in time to readmissions or death nor in emergency visits were found

Demiris et al. (2003) [21]	To examine technical problems that affect the interaction between nurse and patient during virtual visits (VV) and to assess the verbal interaction	Randomized controlled trial	Ten patients with mean age of 78; six patients had congestive heart failure, three had COPD, and one had diabetes-related wounds. 10 nurses from one urban and three rural home care agencies	VV were used for assessing the patient's clinical status, promoting medication and treatment compliance, psychosocial issues, and patient information and education	Technical quality (10-item questionnaire^a^)	One hundred and twenty-two VV were reviewed. Mean duration 21 min. Patients received on average two sessions with 30-minute training. No technical problem in 78 visits. Besides technical issues the VV comprised the following themes: general information, clinical status, psychosocial issues, education, promoting compliance, patient satisfaction, administrative issues, and accessibility. The nurses reported the VV as very useful or useful for patient care and that the majority of the VV would not have been performed better in person

Finkelstein et al. (2004) [22]	To demonstrate quality, clinical usefulness, and patients' satisfaction with home telehealth (HTH) and virtual visits (VV)	Randomized controlled trial	Fifty-three patients with heart failure, COPD, and chronic wounds, mean age 74 and randomized to three groups: (1) standard care + videoconference, (2) standard care, videoconference, and monitoring, or (3) control group receiving standard home care only	VV consisted of two-way audio and video interaction between nurses and patients at home, a web-messaging system, and physiological monitoring system	Telemedicine Perception Questionnaire Home Care Client Satisfaction Instrument	A total of 567 VV were conducted, with 276 by video. Nurses report few technical problems and reported VV useful and time saving for the nurses, but not for the patients. The video/monitoring-group was significantly more positive to HTH after testing for several weeks. Satisfaction with homecare increased for virtual visit subjects. These patients felt safe and that the nurses paid attention to their concerns and met their needs. The VV were found to be time flexible and easy to schedule

Jönsson and Willman (2008) [23]	To compare conventional methods on wound home care with a virtual concept for patients with leg wounds	Quasi-experimental study with a test group and a comparison group. Surveys, field notes, digital photos, and videotaped observations	Ten patients with wounds on lower leg/foot and their responsible nurse. Eight patients and nurses in the control group. Same inclusion criteria, but no broadband access in the control group	The virtual information and consulting concept, *Everyday Learning—Leg Wounds and Their Treatment *consisted of a web application containing advice on nutrition, exercise and wound treatment, and a videophone	Interaction between patient and nurse (22- item questionnaire^a^)	Patients in both groups reported seeing staff caring for them and receiving information and a rapid reply as beneficial. Patients in the test group reported a need of more time to familiarize themselves with the technology. Nurses in the test group found the technology useful for them more than for the patients; it was humane and economical, with the web material as an educational resource. The nurses in the test group had more frequent and longer travel times due to the patients' being in poorer condition than patients in the control group

Van Der Heide et al. (2012) [24]	To establish the effects of a video communication system (CareTV) on loneliness and safety in elderly patients. To evaluate the use of video communication (actual use and expectations) and user satisfaction with CareTV	Longitudinal study with baseline data collection at moment of inclusion, and a follow-up measurement 1 year after inclusion	One hundred and thirty frail elderly clients of home care organizations, mean age of 73 years	The videophone (VP) service of the CareTV was called “Good morning, Good Evening” and included medication service used on a daily basis and an alarm function used less than once per month	The Loneliness Questionnaire Clients feelings of safety (nine-item questionnaire^a^)	Eighty-eight percent of the clients were satisfied with the use of the VP. Technical problems were reported by 12% of the participants. For 63% of the participants, feelings of loneliness decreased significantly (*P* < 0.001). CareTV had no effect on feelings of safety, explained by technical problems and patients experiencing deteriorating health conditions through the study

Mair et al. (2005) [25] UK	To compare the perspectives of patients and providers on telecare encounters via virtual visits used to reduce hospital readmission	Descriptive, evaluation study by the use of log reports and a questionnaire	Twenty-two nurses and 22 patients with COPD. Mean age of 71 years	Videophone intervention to support management of acute exacerbations in COPD patients living at home	Patient and provider perspectives on home telecare (10-item questionnaire^a^)	Nurses completed 150 logs, and patients 145 logs. The patients consistently reported significantly more positive views of the telehealth encounters than their health care providers. Care providers were concerned about the negative effects of telehealth on communication and their ability to assess the patient's medical problems accurately, and they were less comfortable when using the telehealth system

Savolainen et al. (2008) [26]	To evaluate the “Assisting Carers using Telematic Interventions to meet Older peoples' Needs” (ACTION) system	Semistructured interviews and data logging	Eight family users (caregivers and elderly persons), with mean age 73 years. Four professional caregivers	The ACTION-project using information and communication technology to enhance quality of life and independence and reduce social isolation in frail elderly and family at home	n.a.	Average call time was 40 min/month/user; average initiated calls were six per user. Patients reported videophone (VP) reduced loneliness and isolation, assisted with making new social contacts, and increased social activities and safety. Barriers were having enough people to call and technical start-up problems. Caregivers reported the VP created trustful communication; the camera was useful in demonstrating procedures and making assessments. Privacy issues, technical problems, and need for a learning period for the family were a concern

Smith et al. (2007) [27]	To assess technical success, medication self- administration, neuropsychiatric symptoms, and mood burden following use of interactive video technology to monitor medication compliance in persons with mild dementia	Quasi-experimental study with quantitative outcome data from patients. Assessments of medication accuracy and neuropsychiatric conditions. Semistructured interviews with patients and caregivers	Fourteen elderly (aged 80–85) with mild dementia living alone. Eight persons received a video monitoring or a telephone service. Six persons received telephone service or standard unmonitored medication compliance service and acted as control group	Televideo technology to monitor medication compliance	Geriatric Depression Scale Neuropsychiatric Inventory Medication accuracy records	Participants with video and phone monitoring were contacted 4,000 times by nursing assistance services according to their medication schedule. End medication compliance was 81% in the video-monitored group compared to 80% in the phone-group, and 66% in the control. Comparison of compliance from initial to end ratings shows stable compliance in monitored participants, were reduced in unmonitored participants. Rate of change between video and no-monitoring was significantly different (*P* < 0.05). The qualitative interviews revealed that the caregivers experienced less worry and that the technology improved the patients' medication compliance. Other perceived benefits from the technology were prevented relocation to a nursing home and increased social contact

Wade et al. (2009) [28]	To assess the practicality, suitability, safety, and costs of delivering daily home medication management by virtual visits (VV)	Pilot study Semistructured interviews	Nine clients (aged 61–85 years), six of them with dementia and one with memory loss. Four call center nurses	Two participants received Directly Observed Therapy for tuberculosis, 7 receiving general medication management	n.a.	Average length of the VV was nine minutes compared to 19 minutes for an equivalent field visit. One virtual visit was 60% of the cost of a field visit. All clients reported the VV easy to use, and six would continue use. One mentioned flexibility of service as a positive. Nurses reported personal contact, continuity of care, flexibility, and early intervention as advantages

^*^n.a.: not applicable, ^a^scale developed for application in the particular study.
